# A patient derived xenograft model of cervical cancer and cervical dysplasia

**DOI:** 10.1371/journal.pone.0206539

**Published:** 2018-10-26

**Authors:** Luke I. Larmour, Fiona L. Cousins, Julie A. Teague, James A. Deane, Tom W. Jobling, Caroline E. Gargett

**Affiliations:** 1 The Ritchie Centre, Hudson Institute for Medical Research, Clayton, Victoria, Australia; 2 Department of Obstetrics and Gynaecology, Monash University, Clayton, Victoria, Australia; 3 Melbourne Pathology, Collingwood, Australia; Istituto Nazionale Tumori IRCCS Fondazione Pascale, ITALY

## Abstract

**Aim:**

To develop a patient derived xenograft (PDX) model of cervical cancer and cervical dysplasia using the subrenal capsule.

**Methods:**

Cervical cancer (12 Squamous Cell Carcinoma, 1 Adenocarcinoma, 1 Adenosquamous Carcinoma), 7 cervical dysplasia biopsy and normal cervical tissues were transplanted beneath the renal capsule of immunocompromised NOD/SCID/gamma mice. Resulting tumours were harvested and portions serially transplanted into new recipient mice for up to three in vivo passages. Parent and xenograft tumours were examined by immunohistochemistry for p16^INK41^, HPV, and CD-45. Single cell suspensions of mixed mouse and human, or human only cell populations were also transplanted.

**Results:**

The overall engraftment rate for the primary cervical cancer PDX model was 71.4 ±12.5% (n = 14). Tumours maintained morphological, histoarchitecture and immunohistochemical features of the parent tumour, and demonstrated invasiveness into local tissues. Single cell suspensions did not produce tumour growth in this model. Mean length of time (32.4 +/- 3.5 weeks) for the transplanted tissue to generate a tumour in the animal was similar between successive transplantations. Three of four xenografted cervical dysplasia tissues generated microscopic cystic structures resembling dysplastic cervical tissue. Normal cervical tissue (4 of 5 xenografted) also developed microscopic cervical tissue grafts.

**Conclusion:**

The subrenal capsule can be used for a PDX model of human cervical cancer with a good engraftment rate and the ability to model in vivo characteristics of cervical cancer. For the first time we have demonstrated that cervical dysplasia and normal cervical tissue generated microscopic tissues in a PDX model.

## Introduction

Cervical cancer is a leading cause of morbidity and mortality for women worldwide. It is the fourth most common cancer for women globally, with approximately 84% of cases occurring in the developing world [1]. Cervical screening programs have significantly reduced the incidence in developed countries. Early detection and prevention of cervical cancer is based on the existence of a clear premalignant state, cervical dysplasia, and that the Human Papilloma Virus (HPV) is essential to cervical cancer development [2]. Despite this, specific events that convert dysplasia into invasive cancer are unknown. Radiotherapy is the mainstay of treatment for women with advanced disease [3,4], and attempts to find new treatments have been unsuccessful [5]. There is a need for models to further study cervical dysplasia and cancer, and test new therapies.

Due to the established importance of HPV in the development of nearly all cervical cancer in humans, transgenic mouse models have been developed to study oncogenic contributions of various HPV genes *in vivo*. These have elegantly shown that of the two HPV oncoproteins, E7 is more oncogenic than E6 for cervical malignancy. However, these models have limitations. For example, in these transgenic models the induced cervical cancers are estrogen dependant, whereas the contribution of estrogen to human cervical cancer does not appear essential. Further, it does not model human metastatic disease [6]. In addition, there are differences between the cellular mechanisms within cells that differ between mice and humans, for example telomerase activity in adult somatic cells[7]. The differences between human and mouse metabolism affect both tumour behaviour, and drug actions[8]. Hence, models involving human tissues by xenograft are more applicable to human disease.

The difference between murine and human cancers has been observed in other tumour types [9] and hence xenograft models using tissue taken from human cancers have been developed. Immortalised cell lines frequently used in xenograft models have higher engraftment rates compared to primary cell lines but do not represent the full diversity of cell types within a tumour [7]. Unfortunately, the cell culture process irreversibly alters primary tumour cells from their natural phenotype [10,11]. Patient derived xenograft (PDX) models better represent the range of human tumour phenotypes, maintain gene expression patterns of the parent tumour [12], and offer the potential for the future development of mouse “avatars” for human disease and personalised therapies [13]. For example, correlation between tumourigenicity of ovarian cancer xenografts and clinical progression shows their relevance to patient care [14]. PDX models also allow the observation of progressive genetic alterations in cancer samples over time [15]. PDX models have been established for cancers of the colon, stomach, breast, and ovary [16]. Interest in PDX models is increasing, with efforts to standardize model development underway [17]. The Mouse Tumour Biology database at the time of writing does not contain any entries for PDX models of carcinoma of the cervix uteri [17]. However, cervical cancer PDX models have been reported using the subcutaneous and orthotopic (cervical) models. Engraftment rates were 70% at the subcutaneous site, and 48–75% at the orthotopic [18–21]. Higher engraftment rates (up to 95%) have been reported for other tumour types in the sub-renal capsule compared with subcutaneous locations [22,23]. Successful engraftment is essential for a PDX model to become a reliable clinical tool. There are no models for dysplastic or normal cervical tissue. Here we describe a new PDX model for grafting both cervical dysplasia and cervical cancer using the sub-renal capsule location. Additional aims were to determine whether single cell suspensions from cervical cancer produced tumour growth.

## Methods

### Ethics and tissue collection

Ethical approval for the collection of tissue and data from human participants was approved by the Monash Health Human Research and Ethics Committee (13113B). All patients (n = 26) gave written informed consent. Inclusion criteria were women over the age of 18 with a previous diagnosis of cervical cancer by histopathological examination of cytology or biopsy tissue. Fourteen women were recruited. Women underwent examination under anaesthesia for clinical FIGO staging as part of routine clinical care. Tissues were collected during the examination. Seven women, older than 18 years with a confirmed histological diagnosis of Cervical Intraepithelial Neoplasia 3 (CIN3) also participated. Tissue was taken prior to laser ablation of dysplasia, as planned by the treating unit. Tissue samples were also taken from the uteri of five women undergoing hysterectomy for benign indications, as a control cohort. The following clinical data were collected from participants: cervical pathological diagnosis, FIGO stage, age, gravidity, parity, smoking status, number of HPV vaccine doses received, oral contraceptive or hormone use, and medical history. All women were treatment naïve. Blood samples were collected in EDTA tubes and the buffy coat was extracted within twelve hours by centrifugation at 314g for 10 minutes in 0.1M TRIS/EDTA buffer. The cell pellet was resuspended in fresh TRIS/EDTA buffer re-centrifuged, supernatant discarded, and stored at -80°C.

Biopsy tissue was divided into four portions; one immediately frozen in OCT for histology, one fixed in 10% formalin for paraffin sections, one placed in RNAlater (Life Technologies, USA) for 24 hours at 4°C, excess RNAlater decanted and then stored at -80°C and one placed in Dulbecco’s Modified Eagle Medium: Nutrient Mix F-12 (DMEM/F12, Gibco, USA) culture medium containing 10% fetal bovine serum (FBS), 0.5 mg/ml Primocin (Invitrogen, USA) and 1% glutamine on ice until xenotransplantation into a recipient mouse within five hours of collection, although it was up to eight hours for the three of the fourteen cancer samples, and three of seven dysplasia samples.

### Animals

All animal experimentation was approved by the Monash Medical Centre Animal Ethics Committee A (MMCA2013/16), in accordance with guidelines of the National Health and Medical Research Council of Australia.

Female NOD/SCID IL-2R gamma (NSG) mice, 6–14 weeks of age, were obtained from a breeding colony maintained in-house (MMCA2009/25BC). NSG mice have severely impaired immune function, lacking T cells, B cells and Natural Killer cells [24]. Animals were housed in a Specific Pathogen Free barrier facility provided with HEPA filtered air with free access to sterile, standard rodent chow, and sterile water. Environmental enrichment for the animals was provided with tissue paper and cardboard. Anaesthesia was by intraperitoneal ketamine 100 mg/kg (Ceva Animal Health Pty Ltd) and xylazine 10 mg/kg (Troy laboratories Pty Ltd) injection, and analgesia by carprofen 0.5 mg/100 gm (Norbrook Laboratories, Australia) injection subcutaneously. When animal sacrifice was required, euthanasia was performed by either carbon dioxide asphyxiation or cervical dislocation by trained staff.

### Patient derived xenograft procedure

Mice were anaesthetised with intraperitoneal ketamine 100 mg/kg body weight and xylazine 10 mg/kg body weight (both Troy Laboratories, Australia). Mice were placed in the right lateral position and a 2 cm left loin skin incision was made. The peritoneal cavity was entered by an incision made in the abdominal wall overlying the left kidney (Panel A in S1 Fig). The kidney was gently exteriorised, and the renal capsule opened with a dental probe and space opened beneath the kidney capsule with fine forceps. 2–4 pieces (1 mm3) of biopsy tissue chips were inserted in up to four mice/patient sample (Panel B in S1 Fig). The kidney was returned to the abdomen and the skin closed with Michel clips (Fine Science Tools, USA).

Mice were housed for 2–8 months following transplantation until tumour growth was externally obvious and the mice were then euthanized. For the first four samples transplanted, animals were sacrificed at predetermined time points; 4, 12, 24, and 32 weeks, if prior adequate tumour growth was not apparent. This pilot evaluation of tumour size yielded an estimation of rate of growth and the maximum time from transplant euthanasia was set at six months.

At necropsy mice were examined for tumour growth and metastases. Macroscopic tumours were measured by height, width, and length using callipers. Ellipsoid tumour volume was calculated by the formula ½ x length x width x height [25]. Macroscopic tumours were divided into 4 parts for the following; serial retransplantation, RNAlater, OCT, and 10% formalin. Formalin-fixed, paraffin-embedded xenografts were stained with H&E for histopathological confirmation of tissue/tumour growth.

### Retransplantation of PDX tissues

The explant tissue for retransplantation was cut up into small pieces and half was serially transplanted as described above, the remainder dissociated into a single cell suspension as described in S1 Text.

### Immunohistochemistry

Paraffin embedded sections of primary biopsies and xenografts were immunostained with mouse anti-p16 INK4a (Abcam ab54210), mouse anti-HPV (Abcam ab2417), rabbit anti-human cytokeratin 17 (Abcam ab53707), all at 1:100 dilution in 2% FBS/PBS and incubated overnight at 4oC. The antibody used to detect HPV was developed against the BPV L1 product, and has been shown to be reactive against L1 for HPV types 1, 6, 11, 16, 18, and 31 [26]. Mouse anti-human nuclear antibody (Merck-Millipore MAB1281) was incubated overnight at 4°C at a dilution of 1:20, and mouse anti-human CD45 (Invitrogen MHCD4520) at a dilution of 1:50. The secondary antibody used for mouse primary antibodies was biotinylated goat anti-mouse (Vector BA9200) at a dilution of 1:500 incubated at room temperature for thirty minutes. The secondary antibody used for rabbit anti-cytokeratin 17 was goat anti-rabbit IgG F(a,b)2-b at a dilution of 1:500 for 30 minutes. This was followed by streptavidin HRP at 1:200 dilution. Chromogen development was with DAB in stable peroxidase substrate buffer (Thermo Scientific) for five minutes. Dako mouse IgG1 isotype negative control was used for mouse antibodies and rabbit IgG for rabbit antibodies. Slides were examined by bright field microscopy using an Olympus BX10 microscope and images captured with cellSense Standard software version 1.12 (Olympus, Japan). For immunofluorescence PE-conjugated rat anti-mouse CD45 (eBioscience 12-0451-82), was incubated at a concentration of 1:100 for 60 minutes. Nuclear staining was with Hoechst at 1:2000 dilution in PBS for three minutes. Immunofluorescence slides were imaged using a Nikon C1 confocal microscope. Haematoxylin-Eosin stained slides for all harvested tissues and primary biopsies were analysed by an anatomical pathologist (J.A.T.) to confirm diagnoses and the presence or absence of invasive tumour or dysplasia in xenografted tissues.

### Statistical analysis

Microsoft excel version 16.17 was used for maintaining the database. GraphPad Prism Version 6.0 was used for statistical analysis. Demographic data was grouped according to whether a cancer, dysplasia, or normal sample. Tumour growth data was grouped by xenotransplantation number of the graft. Groups were tested for normal distribution with D’Agostino and Pearson normality test. Groups were compared by non-parametric testing with the Wilcoxon signed-rank test and Kruskal-Wallis test followed by Dunn’s post-hoc test. Statistical significance was taken as a p-value of <0.05.

## Results

### Donor demographics

The demographic features of the 26 women recruited for this study are summarised in Table 1. The women with cervical cancer (n = 14) ranged from 28 to 76 years of age, with a median of 48 years. Both age groups of peak incidence (early 30s (n = 4) and >70 years (n = 3) [27]) were represented. Median parity was 2 births (range 1–6). Six (42.8%) were smokers, two (11.7%) had completed the full HPV vaccination protocol (Gardisil, Merck) and one had received a single dose, and two (14.3%) were on the oral contraceptive pill (OCP). All but two of the tumours biopsied were squamous cell carcinomas (SCC), one showing an area of adenocarcinoma-in-situ. The other tumours were a low-grade villous adenocarcinoma and an adenosquamous carcinoma, which also showed Adenocarcinoma-*in-situ*. Most women (n = 13, 64.3%) were FIGO stage 1 at diagnosis. The most advanced case was FIGO stage 3B.

**Table 1:** Patient demographics.

				p value		
	Cervical Carcinoma	Cervical Dysplasia	Normal*	Carcinoma vs Dysplasia	Carcinoma vs normal	Dysplasia vs normal
Number of women	14	7	5			
Age (Median, range)	46, 28–76	32, 24–67	47, 41–49	0.11	>0.99	0.39
Gravidity (Median, range)	3, 1–9	1, 0–4	4, 2–6	0.75	0.62	0.15
Parity (Median, range)	2, 1–6	0, 0–4	2, 1–4	0.23	0.88	>0.99
Smoker (%, Range)	42.8, 21.4–67.4	0, 0–35.4	40, 7.1–76.9	0.15	>0.99	0.44
Yes	6	0	2			
No	8	7	3			
HPV Doses (% full course)	14.3	42		0.28		
3	2	3				
< 3	1	0				
0	11	4				
OCP (%, Range)	14.3,2.5–39.9	0, 0.0–35.4	0, 0.0–43.4	0.77	0.94	>0.99
Yes	2	0	0			
No	12	7	5			

*Diagnosis; 2 fibroids, 2 adenomyosis 1 arteriovenous malformation of uterine wall. Gravidity, no. of pregnancies; Parity, no. of births

The 7 dysplasia samples were from women aged 24–67 years of age; median age was 32 years (Table 1). Half were nulliparous, however parity or gravidity was not significantly different to the cancer group. None were smokers or OCP users, and three had completed full HPV vaccination. All had been previously diagnosed with CIN3.

The 5 normal samples were from women undergoing hysterectomy for benign conditions unrelated to cervical neoplasia. The median age in this group was 47 years. Mean gravidity and parity did not differ from the other groups. 40% were smokers, and none were taking the OCP.

### Development of the subrenal capsule PDX model for cervical cancer

The first sixteen samples were transplanted as part of a pilot phase to determine the optimum time for xenograft growth. S1 Table shows the ellipsoid volume for the xenografts collected from this pilot at pre-determined time points unless adequate tumour growth was achieved. Graft growth was not satisfactory at 4 and 12 weeks, however adequate tumour growth was observed by 24 weeks. The maximum time for graft development was determined to be 24 weeks, unless tumour growth was apparent earlier by palpation.

**Fig 1:**
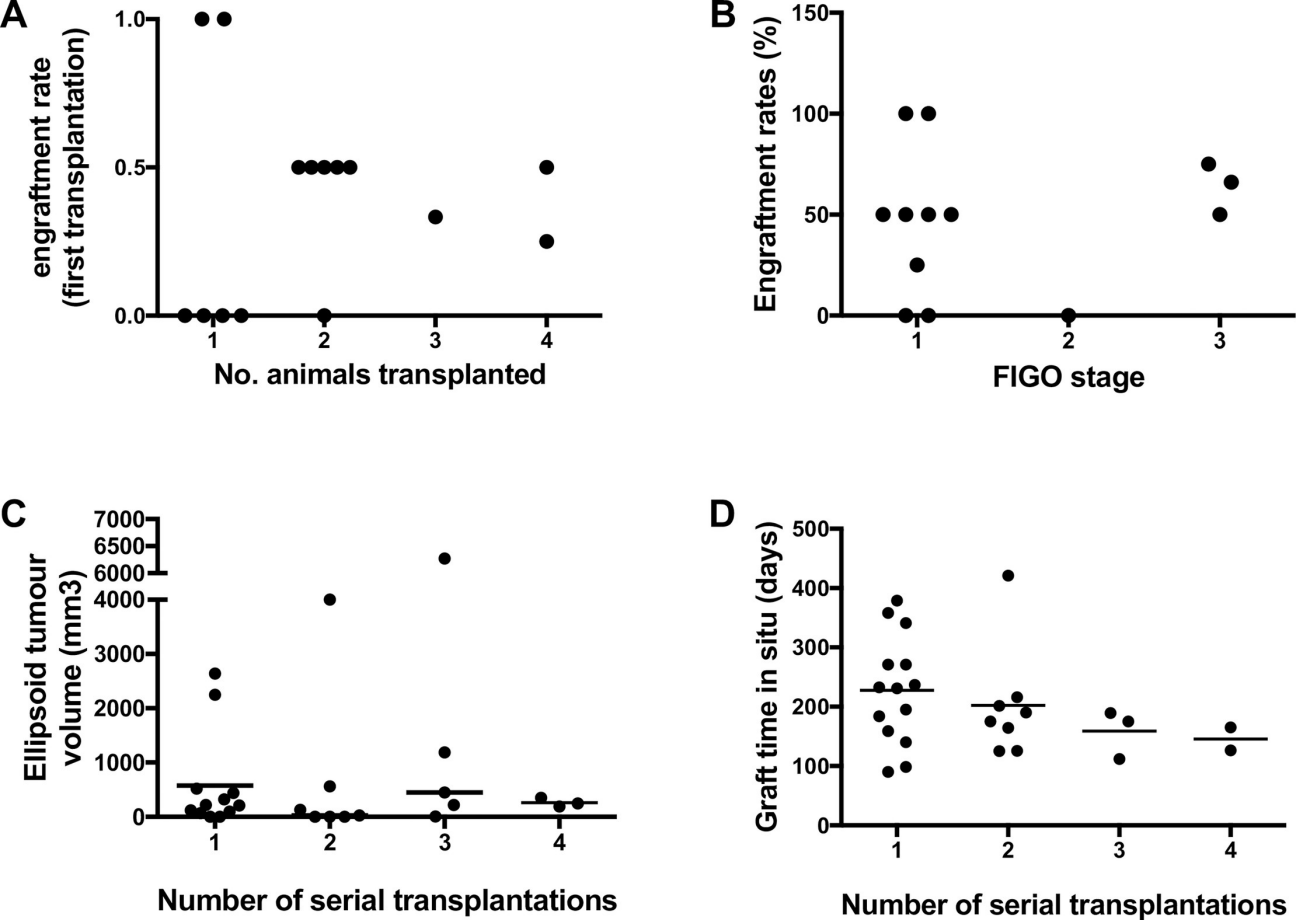
PDX derived cervical tumour engraftment and growth over 4 serial transplantations. Engraftment rate A) in replicate animals for individual patient samples transplanted and B) for each sample according to FIGO stage, C) Tumour volume at harvest at each serial transplantation. D) Length of time between transplantation and cull of animal for each round of serial transplantation for all tumours produced. Bars are medians.

Of the 14 biopsies xenografted, 10 generated harvestable primary tumours resulting in a primary tumour engraftment rate of 71.4 ±12.5% (Table 2). One to four replicate transplantations were performed per sample depending on biopsy size. No difference in mean engraftment rate/sample was observed when grouped by replicate number, however only two of six samples (33%) grafted to one mouse produced tumours (Fig 1A). Only 6 of 94 xenografted mice failed to survive the postoperative period and two more perished in subsequent months from independent causes as necropsy showed no tumour growth.

**Table 2:** Growth characteristics of patient derived cervical cancer xenografts.

Sample	FIGO stage	Histological type	Graft growth		Time in vivo for serial xenografts (days)				Tumour volume of serial xenografts (mm^3^)			
					First	Second	Third	Fourth	First	Second	Third	Fourth
CC1	1B1	CIN3/WD-SCC	Yes		184±18	126±70.5			2250			
CC2		LG villous adenocarcinoma	Yes		271±0	190±0			220	562.5		
CC3		MD SCC	Yes		195	175			324	2		
CC4		MD SCC	Yes		237	125±0			120	1		
CC5		W-MD SCC	Yes		231±87.3				2			
CC6			No		379							
CC7	1B2	WD SCC	Yes		90±57				70			
CC8			No		341							
CC9		MD SCC	Yes		271±0	216±0			210	12.5±12		
CC10	2B	SCC/AIS*	No		232±45.5							
CC11		MD SCC	No		358							
CC12	3A	PD SCC	Yes	140±66.7		165±3.5	175±0	126	2640	128	335±162	192
CC13		MD SCC	Yes	159±42		421			4	98		
CC14	3B	MD SCC	Yes	98.5±28.2		201±1.4	189±26.5	165±0	480±40.0	4000	3729±2541	298±52.5
Mean ± SEM			71.4 ± 12.5%	227±24.3		204±38.4	189.5	145±19.5	736±206	2064	3729	244±52.8

No difference in engraftment rate was observed when comparing stage of cancer at biopsy (Fig 1B), although numbers were small. Mean tumour volume did not increase over subsequent serial transplantations (Fig 1C), nor did the length of time of xenografts across serial transplantations (Fig 1D).

The histology of the tumours produced in the PDX model was generally consistent over subsequent generations of xenografts, with some notable variations from the expected histological grade (Table 3). Six patient samples showed increasing severity over one to three sequential transplantations from dysplasia or well-differentiated squamous cell carcinoma (WD SCC) to a poorly-differentiated variety. Interestingly, the biopsy of CC12 taken directly from the tumour generated a moderate-poorly-differentiated (M-PD SCC) PDX and CIN3. These examples demonstrate the diverse cellular populations preserved by this model that can generate histologically distinct tumour grades. The absence of immune surveillance in NSG mice may allow more rapid disease progression for tumour lines where grade increased.

**Table 3:** Examples showing variation in histological tumour grades between primary tumour biopsy and PDX tumours.

	Biopsy	First	Second	Third	Fourth
**CC1**	WD SCC/CIN3	WD SCC	-	-	-
**CC2**	LG villous carcinoma	LG villous carcinoma	Villiform carcinoma	-	-
**CC3**	SCC/CIN3	MD SCC	-	-	-
**CC4**	MD SCC	-	-	-	-
**CC5**	AIS/CIN3	W-MD SCC	-	-	-
		PD SCC	-	-	-
**CC7**	WD SCC	MD SCC	-	-	-
		PD SCC	-	-	-
**CC9**	MD SCC	MD SCC	MD SCC	-	-
**CC12**	PD SCC/AIS	M-PD SCC	CIN3	M-PD SCC	PD SCC
		M-PD SCC	PD SCC	PD SCC	PD SCC
**CC13**	PD SCC	PD SCC/small cell differentiation	MD SCC	-	-
**CC14**	MD SCC	WD SCC	WD SCC	W-MD SCC	W-MD SCC
		W-MD SCC	-	W-MD SCC	-
		-	-	W-MD SCC	-

Samples shown had two xenografts on first xenograft. -,—no tumour developed. AIS, adenocarcinoma in situ; CIN3, cervical intraepithelial neoplasia 3; WD, well-differentiated; MD, moderately-differentiated; PD, poorly-differentiated; SCC, squamous cell carcinoma.

### Developing a PDX model of cervical cancer using single cell suspensions

Since the purpose of the PDX model was to maintain human cervical cancer tissues over time and expand the tissue without *ex vivo* culture, we examined whether single cell suspensions from dissociated primary xenografts could generate secondary tumours. We compared the capacity of primary xenograft cell suspensions (10^6^ cells) with and without removal of mouse fibroblasts to examine the contribution of mouse stroma to the engraftment process. Several primary xenografts yielded >20 million human cells. However, xenografting doses of 10^6^ cells/kidney failed to generate tumours, irrespective of the presence of mouse cells. In contrast, xenografting tissue chips (1mm^3^) yielded good tumour growth for up to three passages. Ability to re-engraft was limited by the size of the harvested xenograft. The optimal period for reliably generating tumours which provided sufficient tissue for characterisation and re-transplantation was approximately six months per serial transplantation.

### Serial cervical cancer xenografts recapitulates marker expression patterns of parental tumours

**Fig 2:**
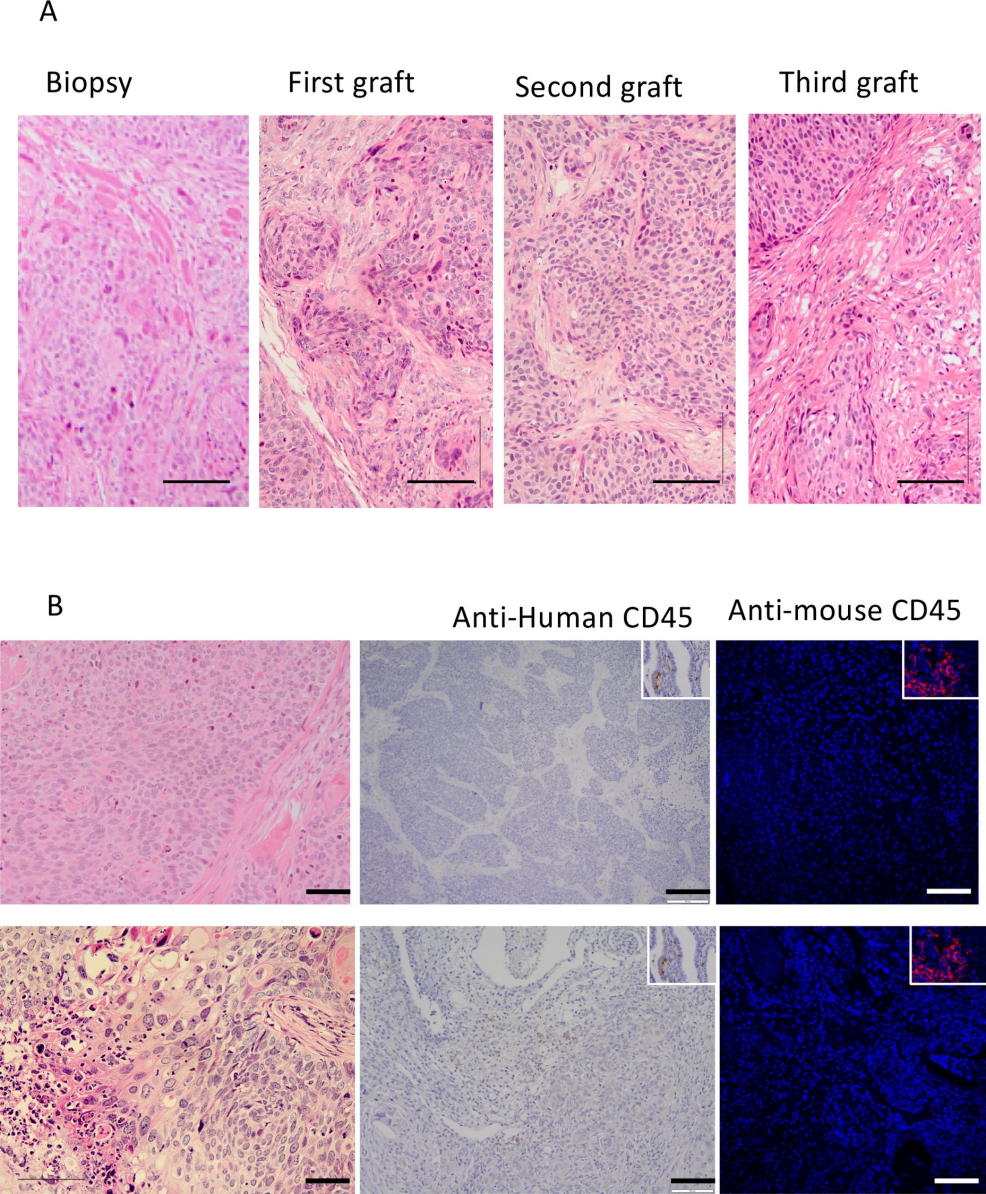
Morphology of serially transplanted cervical cancer PDXs. A) representative example of an H&E stained cervical squamous cell carcinoma sample showing morphology of the tumour biopsy, primary, secondary and tertiary PDXs. B) Typical examples of negative staining for anti-human CD45 staining (second column), and anti-mouse CD45 (third column). Insets show examples of CD45 positive staining in human cervix biopsies and mouse kidney Scale bars 50 μm.

**Fig 3:**
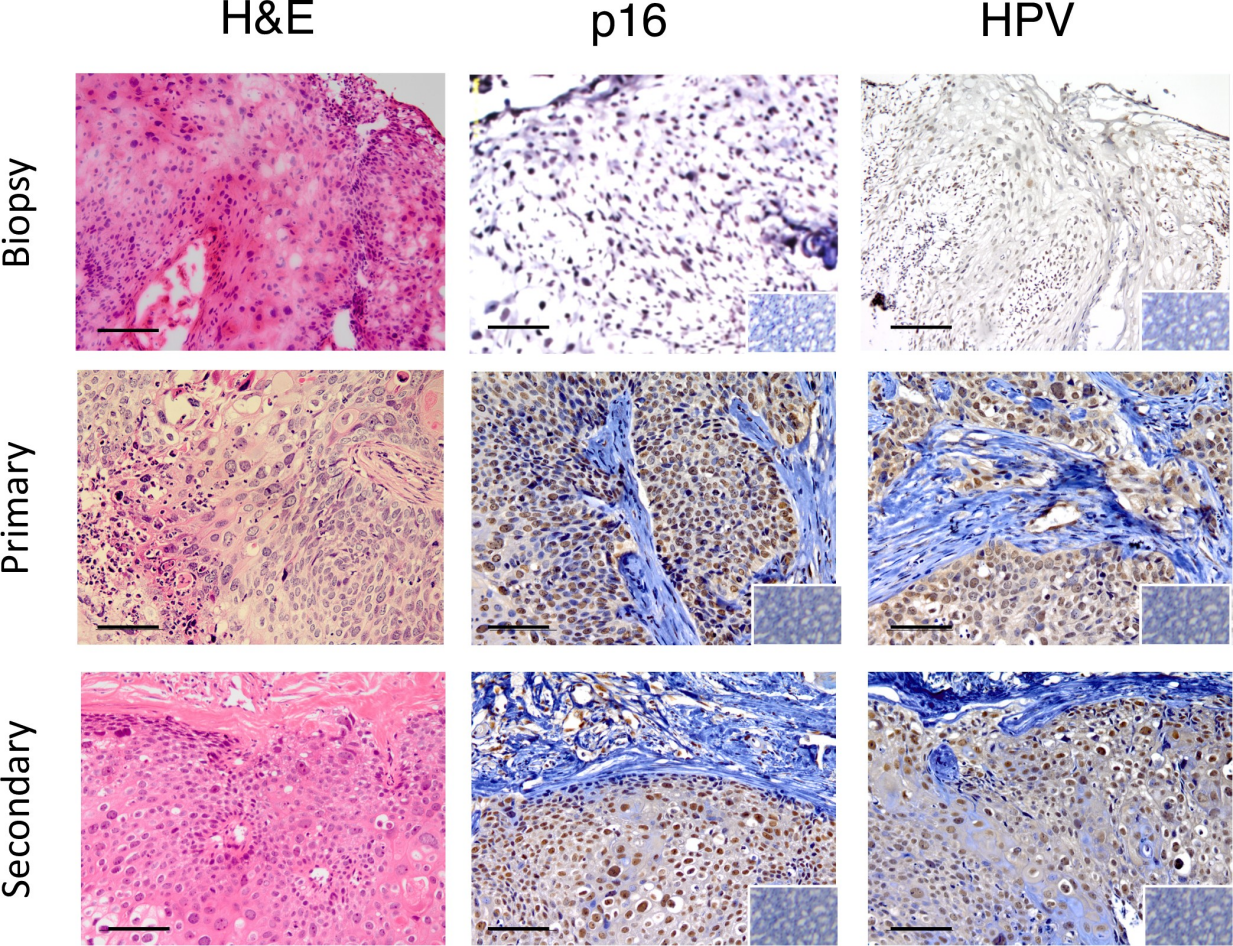
Immunohistological features of serially transplanted cervical cancer PDXs. Representative example of a PDX showing comparable histology (H&E) and immunoreactivity for diagnostic markers of cervical cancer in the primary biopsy and primary and secondary PDXs. Columns 2 and 3 show staining patterns for p16^INK4a^ (brown nuclear staining), HPV (brown nuclear and cytoplasmic immunostaining). Insets, isotype IgG control showing negative immunostaining. Scale bars 10 μm.

Morphological features by H&E staining were maintained between the parent tumour biopsy and subsequent xenograft explants (Fig 2A). Nests of cells with mitotic nuclei were observed and areas with similar patterns of collagen deposition in serially transplanted xenografts and the primary biopsy (Fig 2A). Similar immunostaining patterns for p16INK4a and HPV between the primary tumour and subsequent xenograft explants were observed (Fig 3). Widespread nuclear staining for p16^INK4a^ was maintained between parent and graft. Nests of cells, or in some case sporadic cells, showed cytoplasmic staining for HPV (Fig 3). Xenograft samples showed negative staining for both human CD45 antibody (Fig 2B), indicating a lack of human or mouse leukocytes, confirming that the xenografts are neither transplanted human, nor virally induced murine lymphoma as have been described in other models [17,28,29]. Local invasion of the murine kidney and into the peritoneal cavity by the xenograft was observed in four of eight patient samples yielding tumour growth. Only one case of peritoneal metastasis was observed (CC5).

### Developing a PDX model for cervical dysplasia

**Fig 4:**
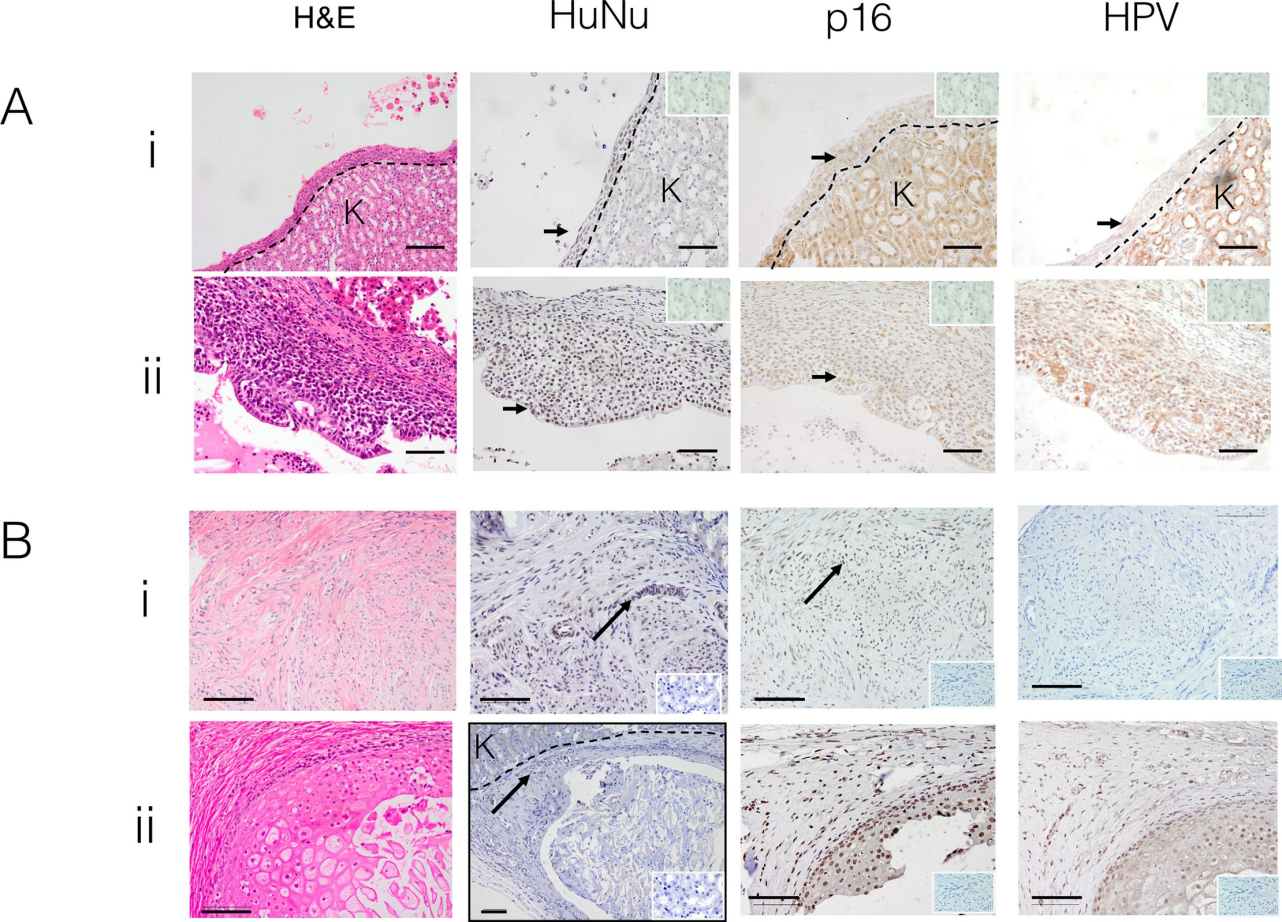
Patient-derived xenografts from cervical dysplasia and normal cervical tissue. A) Two representative examples of the cystic structures formed from cervical dysplasia xenografts. H&E (first column) and immunohistochemical staining for human nuclei antibody (brown nuclei), human p16^INK4a^ (brown nuclear staining), HPV (brown cytoplasmic staining), Insets, IgG isotype negative controls. Scale bars; 50 μm B) **normal cervical tissue xenografts**. Two representative examples of normal cervix after 6 months under the renal capsule of NSG mice, showing (i) cervical stromal tissue and (ii) cervical squamous epithelium. H&E, anti-human nuclear (brown nuclei), human p16^INK4a^ (brown nuclei) and HPV immunostaining of harvested xenografts. Note that unexpectedly, HPV is present in the second sample. Arrows indicate examples of positively stained nuclei. Dotted line; border with mouse kidney. K marks kidney. Insets show IgG isotype controls. Scale bars 10 μm.

Seven dysplasia samples were transplanted as described above. At necropsy, no obvious macroscopic tumour growth was observed. However, microscopic examination of the kidney demonstrated epithelial-lined cystic structures in 3 of 7 patient samples (Fig 4A). The lining epithelium immunostained with human nuclei antibody, indicating the cells were of human origin (Fig 4A). The epithelium in 2 of 3 cysts were positive for p16^INK4a^, with patchy HPV staining (Fig 4A). This data suggests that these two cysts represent persistent survival and growth of cervical dysplasia tissue xenografted beneath the renal capsule.

We also examined whether normal cervical tissue survived transplantation under the kidney capsule. Four of five samples survived for four months under the kidney capsule, resulting in microscopic growths similar to the cervical dysplasia samples (Fig 4B), however the tissue that persisted appeared to be stroma rather than the epithelium from which cervical squamous carcinoma arises. These xenografts immunostained for anti-human nuclear antibody and suggest growth of normal cervical tissue in an animal model for the first time. Sporadic p16^INK4a^ immunostaining was observed in all samples. HPV staining was not seen in the stromal cervical xenograft tissue (n = 3) (Fig 4B), consistent with its stromal appearance and stromal tissue is not typically infected by HPV. However, in a single sample typical squamous epithelial cells were observed, and these were HVP positive (Fig 4B ii). This sample may be a previously undetected case of dysplasia, however no dysplastic histological features were seen.

## Discussion

The main finding of this study was our demonstration for the first time that fresh cervical cancer, cervical dysplasia, and normal cervical tissues can grow beneath the renal capsule of highly immunocompromised mice. Tumours from cervical cancer xenografts recapitulated parent tumour architecture and immunohistochemical profiles for p16 and HPV for at least 3 passages *in vivo*. Generated tumours were invasive but metastases were rare in our model. We demonstrated for the first time that cervical dysplasia tissues generate microscopic cystic growth under the murine renal capsule showing features expected of dysplasia for key markers of cervical cancer [30]. Similarly, we demonstrated for the first time that the NSG renal capsule permitted the survival and microscopic growth of normal human cervical tissue *in vivo*. Human cervical cancer tissue grew slowly, requiring 6 months to generate adequate tumours for serial transplantation and characterisation. The renal capsule site provides a unique approach for the biological study of cervical dysplasia conversion into cervical cancer, albeit a lengthy process. Key tumour characteristics preserved in this model were histological morphology, and cervical cancer markers p16^INK4a^ and HPV. The sub-renal capsule is highly conducive to transplantation of xenograft samples and opens possibilities for studying the natural history of cervical neoplasia or for assessing new treatments. The microscopic size of the dysplasia and normal tissue grafts may hinder the clinical applicability of these aspects of this model.

The finding that two of four dysplastic samples resulted in microscopic growth of human tissue with immunohistochemical features consistent with cervical dysplasia is significant. To our knowledge this is the first time that dysplastic tissue has been intentionally cultivated in a xenograft model. Growth of premalignant cells has rarely been described, and reported only once as a serendipitous finding in a prostate cancer PDX model [22]. These cells have subtler variations from normal and are much less tumorigenic. Importantly our PDX model offers the opportunity to examine the development of cervical cancer from its premalignant state. There is potential for dysplastic tissue growing in a PDX model to be harvested for use in future studies, although the small size of the xenograft will make this technically challenging. Lesions that eventually progress to carcinoma after multiple passages *in vivo* could be examined to determine changes at a molecular level that allowed the dysplastic cells to become invasive. In addition, the successful growth of microscopic normal cervical tissue in a PDX model is described here for the first time.

PDX models likely select more tumorigenic cell subpopulations within tumours with capacity to thrive in the murine milieu and respond to murine growth factors, particularly in animals without a competent host immunity [7]. Our sub-renal capsule PDX model shows this occurred for the majority of samples. A key difference between this model and other cervical cancer PDX models is the mouse breed. Nude or Scid mouse models have been used previously [18,20,21]. However, we used NSG mice, which are more profoundly immunosuppressed, lacking natural killer (NK) cell immunity [24]. NK cells mediate their function through Major Histocompatability Complex (MHC) recognition, and destroy non-self cells [31]. Without this surveillance, neoplastic, dysplastic, and normal xenografted tissue growth was enabled, a major advantage with our model, although only cancer biopsy samples produced large cell masses. The inability to recognise MHC molecules likely greatly improves the engraftment of human cells, however, the study of tumour cell interactions with host innate immunity is a limitation of this model.

A lower engraftment rate was obtained for sub-renal capsule xenografts compared with prostate and endometrial cancers [22,23] for reasons that remain unclear. A few samples had a longer time to engraftment, which would potentially lower engraftment success. These were earlier samples when skill acquisition necessitated a longer procedural duration. However, these earlier PDX did not have a lower engraftment rate. Although published PDX models of non-cervical cancers have allowed even longer windows for transplantation of up to 24 hours[32], future applications of this model should aim for engraftment of freshly obtained samples within the first few hours to ensure maximum tissue viability. The engraftment rate of this model was comparable to a subcuticular PDX model for cervical cancer of 70% [18], and the recently described orthotopic PDX model at 75% [20] suggesting engraftment rates are tissue and tumour specific. At the commencement of this study the highest rate achieved by an orthotopic PDX model of cervical cancer was only 48% [18–21]. Another group has since published a PDX model using the sub-renal capsule as transplantation site and achieved an engraftment rate of 66.7%, which is comparable to our own rate [33].

PDX models require the combination of high engraftment rates, technical ease, and maintenance of *in vivo* tumour characteristics. Subcutaneous models provide easy access to the xenograft site for monitoring tumour growth [7], but do not accurately model *in vivo* tumour behaviour as they become encapsulated [18]. Further, direct comparison between the subcutaneous and orthotopic sites for the same patient sample showed that orthotopic transplantation, but not subcutaneous, mimics the metastatic pattern observed in the patient [20,34]. A high engraftment rate is essential for clinical application. Our study suggests that engraftment success can be maximised by engrafting at least 2 animals for each sample. Our finding that subsequent passages of PDX grafts sometimes yielded varied tumour grades suggests that multiple replicate transplantations of each sample should improve preservation of cell population diversity of a patient’s tumour. Hence, to ensure the best use of this resource in a potential future ‘patient avatar’ situation at least two animals should be transplanted for each patient sample.

No growth was achieved from the transplanted cervical cancer cell suspensions, despite a previous application of cell suspensions to orthotopic xenografting, [21]. The inability of cervical cancer cell suspensions to produce tumours compared to other reproductive tract tumours such as endometrial carcinoma [23] may be due to the extensive collagen laid down by SCC of the cervix. Harsher digestion is required which damages cells by stripping adhesion molecules on the tumour cells. Cell suspensions from stomach cancer xenografted to the orthotopic location yielded lower metastatic rates than tissue pieces surgically grafted to that location [16]. This may also be due to better preservation of multiple cell populations required for tumour growth and that non-tumour human cells provide growth factors and signalling mechanisms that improve cancer cell survival and proliferation. Alternatively, transplantation of tumour pieces may better preserve cell cues by maintaining the tumour microenvironment and microarchitecture.

A limitation of the sub-renal capsule model is difficulty monitoring tumour growth. As the tumour grows beneath the kidney surface, tracking of tumour size at early stages is difficult compared with the subcutaneous site. Tumour growth is often not apparent until the tumour is of considerable size. We used palpation of the flank, in combination with a pilot phase to assess lesion size at necropsy, to assess for tumour growth. The pilot phase also suffers from the limitation of being conducted through the skill acquisition phase, which may have reduced the success of some engraftments. The difficulty in creating a single-cell suspension inhibited our ability to transfect the cells with luciferase for bioluminescence imaging. The model could be strengthened, however, with the addition of other modalities of non-invasive imaging such as ultrasound or MRI. Future applications of this model should make use of these technologies to monitor the variability of individual tumour growth rates that are a feature of PDX models.

However, sub-renal capsule transplantation overcomes problems of xenograft encapsulation and low rates of metastasis compared to the subcutaneous site. The long latency time of this model is not unexpected or unique to this particular PDX model [15]. It does, however present difficulties to the clinical application of PDX models as “patient avatars” [13]. In this concept, the PDX animal bearing the graft of an individual patient could undergo sample treatments to determine the optimal regimen for the donor patient. Cancer treatments cannot wait six months, suggesting that the best application of this model may be for recurrent or resistant disease once standard therapies fail.

## Conclusion

The subrenal capsule provides an excellent alternate model for generating PDX for the study of tumour progression and evaluating therapies in cervical cancer. The ability to detect cervical dysplasia and normal cervical tissue cells is novel and provides models for the study of tumour initiation and progression.

## Supporting information

S1 Fig Images of PDX procedure and graft on kidney. A) photograph showing the position of the animal, with the left kidney exteriorised through the abdominal wall incision, B) post-mortem kidney specimen showing the location of xenograft as indicated by the arrow showing a 4 mm long tumour on the kidney surface.
(TIF)

S1 Text Supplementary methods. Method used to create single cell suspensions from PDX explants.
(DOCX)

S1 Table Xenograft growth data for pilot phase. (DOCX)
