# Supplementary figures and images for: A patient derived xenograft model of cervical cancer and cervical dysplasia
Source: PLoS One. 2018 Oct 26;13(10):e0206539. doi: 10.1371/journal.pone.0206539 (PMC6203389; doi:10.1371/journal.pone.0206539)

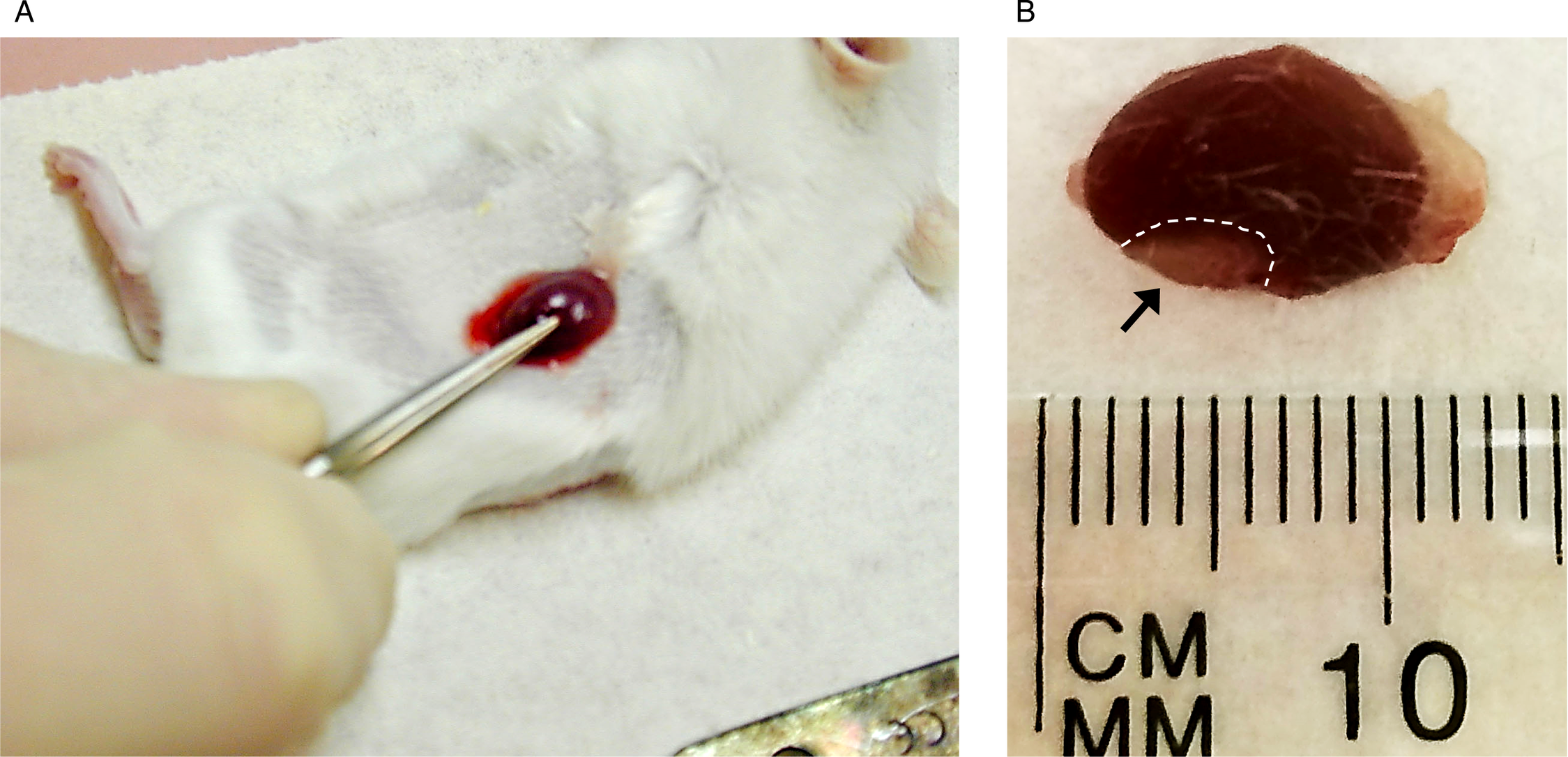

Supplement: S1 Fig — A) photograph showing the position of the animal, with the left kidney exteriorised through the abdominal wall incision, B) post-mortem kidney specimen showing the location of xenograft as indicated by the arrow showing a 4 mm long tumour on the kidney surface. (TIF) [file pone.0206539.s001.tif]
