# Supplementary material for: A patient derived xenograft model of cervical cancer and cervical dysplasia
Source: PLoS One. 2018 Oct 26;13(10):e0206539. doi: 10.1371/journal.pone.0206539 (PMC6203389; doi:10.1371/journal.pone.0206539)
Supplement: S1 Text — Method used to create single cell suspensions from PDX explants. (DOCX) [file pone.0206539.s002.docx]

# S1 Text, Supplementary methods

## Creation of single cell suspension

Tissue was dissociated physically by mincing and then digested with 5 mg/ml collagenase 1 (Worthington Biochemical Corporation, USA), 80 μg/ml DNAase (Invitrogen, USA) and 5 mM glucose (Sigma Chemical Co, USA) in PBS in a total volume of 10 ml for two to three hours at 37^o^C with constant agitation and intermittent trituration until single cell suspensions were achieved. The collagenase 1 solution was refreshed half way through digestion. The resulting cell suspension was centrifuged at 264 g at 4^o^C for five minutes and the supernatant removed. The pellet was resuspended and cell count performed. Two aliquots containing one million cells each were removed to create a mixed human and mouse cell suspension. After repeat centrifugation, the pellet of cells was resuspended in 100 μl 0.5% FBS/PBS per 10^6^ cells. PE conjugated anti-mouse H-2K^d^ antibody (IgG2a isotype) (BD Pharmingen, USA) (5 μl) was added per 10^6^ cells and incubated on ice in the dark for 30 minutes to label contaminating mouse cells. The cells were then incubated with 8 x 10^6^ PE-conjugated magnetic microbeads (Miltenyi, Germany) ) on ice in the dark. The cells were then passed through an MS column (Miltenyi) and placed in a magnet (Miltenyi) to collect the mouse cells and the filtrate containing human cells collected. A cell count was performed and the sample was divided into one million cell aliquots. These formed the human only cell suspensions, which were centrifuged and the cell pellets then embedded in 10 μl fibrin gel (10 μl of fibrinogen (Merck, USA) in bench media 50 mg/ml with 1% thrombin (Sigma, USA) and transplanted beneath the renal capsule of the NSG mice.
