# Supplementary material for: A patient derived xenograft model of cervical cancer and cervical dysplasia
Source: PLoS One. 2018 Oct 26;13(10):e0206539. doi: 10.1371/journal.pone.0206539 (PMC6203389; doi:10.1371/journal.pone.0206539)
Supplement: S1 Table — (DOCX) [file pone.0206539.s003.docx]

**S1 Table. Xenograft growth data for pilot phase**

| **Time point (weeks)** | 4 | 12 | 24 | 32 |
| --- | --- | --- | --- | --- |
| **Ellipsoid volume of graft (mm^3^)** | 0 | 0 | 70 | 2 |
|  | 0 | 0 | 440 | 0 |
|  | 0 | nd | 520 | 2640 |
|  | 0 | nd | 0 | 0 |
|  | 0 | nd | 0 | nd |
|  | nd | nd | 4 | nd |

nd, not done
